# Informing primary care reform in Greece: patient expectations and experiences (the QUALICOPC study)

**DOI:** 10.1186/s12913-017-2189-0

**Published:** 2017-04-05

**Authors:** Christos Lionis, Sophia Papadakis, Chrysanthi Tatsi, Antonis Bertsias, George Duijker, Prodromos-Bodosakis Mekouris, Wienke Boerma, Willemijn Schäfer, on behalf of the Greek QUALICOPC team, Ioannis Dakoronias, Sah Hamid Alexandros, Aristofanes Paganas, Dimitrios Ellinas, Vesela Polyzou, Ioannis Lentzas

**Affiliations:** 1 Faculty of Medicine, University of Crete, Heraklion, Crete Greece; 2 Division of Prevention and Rehabilitation, University of Ottawa Heart Institute, Ottawa, ON Canada; 3 The Greek Association of General Practitioners (ELEGEIA), Thessaloniki, Greece; 4 Netherlands Institute for Health Services Research (NIVEL), Utrecht, Netherlands

**Keywords:** Primary care, Quality, Patient-centered care, Greece, QUALICOPC, Cross sectional

## Abstract

**Background:**

Primary health care is the cornerstone of a high quality health care system. Greece has been actively attempting to reform health care services in order to improve heath outcomes and reduce health care spending. Patient-centered approaches to health care delivery have been increasingly acknowledged for their value informing quality improvement activities. This paper reports the quality of primary health care services in Greece as perceived by patients and aspects of health care delivery that are valued by patients.

**Methods:**

This study was conducted as part of the Quality and Costs of Primary Care in Europe (QUALICOPC) study. A cross-sectional sample of patients were recruited from general practitioner’s offices in Greece and surveyed. Patients rated five features of person-focused primary care: accessibility; continuity and coordination; comprehensiveness; patient activation; and doctor–patient communication. One tenth of the patients ranked the importance of each feature on a scale of one to four, and nine tenths of patients scored their experiences of care received. Comparisons were made between patients with and without chronic disease.

**Results:**

The sample included 220 general practitioners from both public and private sector. A total of 1964 patients that completed the experience questionnaire and 219 patients that completed the patient values questionnaire were analyzed. Patients overall report a positive experiences with the general practice they visited. Several gaps were identified in particular in terms of wait times for appointments, general practitioner access to patient medical history, delivery of preventative services, patient involvement in decision-making. Patients with chronic disease report better experience than respondents without a chronic condition, however these patient groups report the same values in terms of qualities of the primary care system that are important to them.

**Conclusions:**

Data gathered may be used to improve the quality of primary health care services in Greece through an increased focus on patient-centered approaches. Our study has identified several gaps as well as factors within the primary care health system that patient’s perceive as most important which can be used to prioritize quality improvement activities, especially within the austerity period. Study findings may also have application to other countries with similar context and infrastructure.

## Background

Many European countries share the goal of establishing a strong primary health care (PHC) system as part of their health care system [1]. There is good evidence supporting the importance of PHC in reducing healthcare spending and health disparities [1–3]. Over the past several years, Greece has been actively attempting to improve health care services with a focus on strengthening PHC [4]. The austerity period, which has been in place in Greece for several years, has had a significant impact on the size, structure, quality and effectiveness of health care services [4].

Internationally, patient satisfaction and patient-centered approaches to health care delivery have gained an important place in informing health care reform and quality health care delivery [5, 6]. Meeting the expectations of the population through patient-centered health care delivery is one of the valued indicators of health system functioning [1, 7]. Several studies have demonstrated that a positive relationship between general practitioners (GPs) and their patients is associated with reduced health care spending [8] and there is good evidence to show that the relationship between patients and PHC professionals can be reinforced by improved communication during practice visits [9–11] The key elements of patient-centered approaches are: eliciting patient perspectives, responding to patient concerns, giving information; partnership building; engaging the patient in participatory decision-making; and developing a follow-up health care plan together [12–14].

In Greece, PHC is delivered through a combination of publically funded state health services and by private Family Physicians/GPs. The public service is delivered through Health Centres, which are accessible 24 hours a day, 7 days a week, and located primarily in rural areas. GPs provide their services on a fee-for-service basis in privately owned offices [4, 15].

The public PHC services can provide a number of services including diagnostic, curative and preventive health, facilitating multi-disciplinary team of professionals such as GPs, pediatricians, microbiologists, dentists, midwifes, radiologists, and nursing personnel. On the other hand, patients are free to choose whether they will visit a public PHC service or a specialist from the private sector. Greek patients often have unequal access to standard PHC services, depending on factors such as geographical accessibility and ability to pay for a private physician. The PHC system in Greece has reported low performance in several areas of quality-based care [4, 16]. A recently published paper of Schäfer et al. reported on data from the Quality and Costs of Primary Care in Europe (QUALICOPC), which examined quality indicators as perceived by patients in 34 countries including Greece [17]. Five dimensions of person-focused primary care have been assessed by the QUALICOPC project: accessibility, continuity and coordination, comprehensiveness, patient activation, and doctor–patient communication [1, 18]. The paper clearly highlighted the tail position of the Greek health care system as perceived by patients, in particular in the areas of accessibility, continuity and patient involvement [17]. These results validate the urgency of continued efforts to reform PHC in Greece and it is considered as a high priority for a country that has been seriously damaged by the austerity period.

Motivated by these alarming observations this paper sought to conduct a more in-depth exploration of the quality of PHC services in Greece as assessed by patients and explores aspects of health care delivery that are valued by patients. Comparisons are also made between patients with and without chronic disease as we hypothesize experiences will differ across these patient sub-groups given the acute care orientation of PHC services in Greece. Finally, we reflect on possible solutions that might inform health care reform activities moving forward. The Clinic of Social and Family Medicine at the University of Crete jointly with the Greek Association of General Practitioners (ELEGEIA) were responsible for the implementation of the QUALICOPC project in Greece. Their main responsibilities included the selection of GP practices, the recruitment of GPs serving in all healthcare centres located in all seven health regions of Greece. Additionally they were responsible for the distribution of the questionnaires and data collection, storage, and analysis.

## Methods

### Setting and design

A cross-sectional survey was conducted of both GPs and a sample of patients from their practice. We aimed to sample one GP from each of the 218 public PHC centers located in the seven health care regions in Greece. A GP-coordinator was identified in each of the health regions to assist with local recruitment activities. The Clinic of Social and Family Medicine, with the assistance of the regional coordinators and ELEGEIA sent invitations to a list of 300 GPs working in the 218 public PHC centres with the aim was to recruit one GP per PHC centre. A total of 203 GPs accepted participation while 15 denied participation, thus 15 (6.9%) PHC centres were not represented in the sample. If more than one GP expressed interest in participation from the same health centre, the first person to respond was included in the sample. Apart from the sample of GPs serving in the public sector, we invited 25 GPs serving in the private sector. They were contacted alphabetically from an available list of 130 GPs eligible serving in the private sector located in the 5th and 7th health regions of Greece and 17 (68%) accepted participation. From each GPs practice a sample of 10 consecutive patients from their practice was surveyed. Written informed consent was obtained from all patients and participant responses remained anonymous. Ethical approval was received from Bioethical Committee of the University General Hospital of Heraklion (number protocol: 7655/7-7-2011).

### Data collection

Study data collection activities occurred between February 2011 and November 2012. Patient recruitment took place between 8:00 am and 15:00 pm in order to capture a wide range of service users. A trained field worker over saw data collection activities at the clinic. Field workers invited consecutive patients who had a face-to-face consultation with the GP and who were over the age of 18 years, and able to speak and read Greek were surveyed, until a sample of 10 questionnaires per practice was achieved. Data was collected at the end of the consultation with the GP. All participants completed a informed consent. Nine tenths of participants completed the patient experience questionnaire and one tenth completed the patient values questionnaire. Details of the QUALICOPC study protocol have been published elsewhere [1, 17, 18].

### Measures

The QUALICOPC Patient Experiences and Patient Values Questionnaires were used to collect data. The questionnaires were developed by the QUALICOPC Consortium and are based on PHAMEU framework [1]. Patients rated the five dimensions of person-focused primary care: accessibility, continuity and coordination, comprehensiveness, patient activation, and doctor–patient communication [1, 18]. The Patient Experience Questionnaire asked patients to indicate whether they agreed with the statement by selecting ‘yes’ or ‘no’ answers. The Patient Values Questionnaire asked patients to rank the importance of each of the statements contained in the patient experience survey using a 1–4 scale, 1 (not important) to 4 (very important). Socio-demographic data (age, gender, employment status), presence of chronic disease(s) (‘Do you have a longstanding disease or condition such as high blood pressure, diabetes, depression, asthma or another longstanding condition?’) and overall health status (‘How would you describe your health status in general?’) were collected from all participants. The English version of the questionnaires were translated into Greek using a forth-and back translation procedure.

### Statistical analysis

Characteristics of participants were summarized using descriptive statistics. Univariate comparisons were performed for patients with and without chronic disease using Pearson’s chi-squared test for categorical data. Patient responses for the values survey were recoded so that 1 = very important and 0 = all other responses. We choose to focus the analysis on the categorization of values as “very important” due to the ceiling effect that was seen when positive responses (i.e. “important” and “very important”) were combined. Missing data was minimal and was not replaced. For each variable missing values are reported in the tables. All analyses were conducted using the Statistical Package for Social Sciences (SPSS) Version 21.

## Results

Two-hundred and twenty GPs within the seven health regions agreed to participate in the study. A total of 2220 patients completed study questionnaires (10 per GP practice); however 37 surveys were not legible and were excluded from the analysis. In total 2183 patients were included in the analysis (1964 patients who completed the Patient Experience Questionnaire and 219 patients completed the Patient Values Questionnaire). The primary reason for ineligibility was the inability to read/write in Greek.

### Demographic characteristics

Table 1 presents the socio-demographic characteristics of patients sampled. Almost 90% of participants were living with other adults and had children. Close to one third, reported a household income that was below average. Almost, forty-one percent of respondents to the Patient Experience Questionnaire and 53.7% of respondents to the Patient Values Questionnaire reported the presence of a chronic disease.

**Table 1:** Socio-demographic characteristics of respondents to Patient Experience and Patient Values questionnaires

Variable	Responders patient experience survey *n* = 1964	Responders patient values survey *n* = 219
Mean Age, years (SD)	52.7 (±16.5)	51.9 (±14.5)
	n (%)	n (%)
Sex^a^		
Male	863 (44.1%)	93 (42.5%)
Female	1093 (55.9%)	126 (57.5%)
Were born in Greece?^b^		
Yes	1867 (95.4%)	215 (98.2%)
No	89 (4.6%)	4 (1.8%)
Other adults living in your household^c^		
Yes	1729 (88.3%)	195 (89.4%)
No	229 (11.7%)	23 (10.6%)
Children in your household?^d^		
Yes	1243 (63.4%)	84 (38.7%)
No	718 (36.6%)	133 (61.3%)
Years of formal education^e^		
0–9 years	721 (37.0%)	54 (25.1%)
9–12 years	654 (33.6%)	78 (36.3%)
>12 years	573 (29.4%)	83 (38.6%)
Current employment status^f^		
Employed	827 (42.3%)	121 (55.3%)
Student	89 (4.5%)	10 (4.6%)
Unemployment	168 (8.6%)	20 (9.1%)
Retired	590 (30.2%)	49 (22.4%)
Other	286 (14.7%)	14 (8.7%)
Household income^g^		
Below average	587 (29.9%)	43 (19.6%)
Around average	1245 (63.5%)	154 (70.3%)
Above average	128 (6.5%)	22 (10%)
Presence of chronic illness^h^		
Yes	792 (40.6%)	117 (53.7%)
No	1158 (59.4%)	101 (46.3%)

*SD* standard deviation

Patient Experience Survey: ^a^ *n* = 1956; ^b^ *n* = 1956; ^c^ *n* = 1958; ^d^ *n* = 1961; ^e^ *n* = 1948; ^f^ *n* = 1960; ^g^ *n* = 1960; ^h^ *n* = 1950

Patient Values Survey: ^a^ *n* = 219,; ^b^ *n* = 219; ^b^ *n* = 219; ^c^ *n* = 218; ^d^ *n* = 217; ^e^ *n* = 215; ^f^ *n* = 219; ^g^ *n* = 219; ^h^ *n* = 218

### Health status and primary care service use patterns

Tables 2 and 3 present information on the health status of patients and PHC service use patterns for the overall sample and those with and without a chronic illness. Fifty nine percent of participants described their health status as good or very good. As would be expected individuals who did not have a diagnosed chronic disease reported significantly better health status than individuals with a chronic illness. Among patients sampled, 10.8% of patients with a chronic disease reported not having a personal physician compared to 31.9% of patients without a chronic disease. During the past 6 months, one in four participants consulting a GP more than 5 times in the previous 12-months. Among patients with chronic illness, the proportion of patients with 5 or more consults increased to 38% (*p* < 0.0001). The main reason for PHC consult was for a prescription (44.9%) and the majority of patients seen for prescriptions were patients with chronic illness (61.8%).

**Table 2:** Participant health status, frequency of GP visit, and reason for current visit overall and by presence of a chronic disease

Variable	Total n (%)	Patients with chronic disease n (%)^a^	Patients without chronic disease n (%)^a^	*p*-value
How would you describe your health status in general:^b^				<0.0001
Very good	409 (20.9%)	52 (4.5%)	353 (44.9%)	
Good	750 (38.4%)	428 (37.1%)	317 (40.3%)	
Satisfactory	617 (31.4%)	502 (43.5%)	111 (14.1%)	
Poor	178 (9.1%)	171 (14.8%)	6 (0.8%)	
Do you have your own personal physician who you consult when you are ill?^c^				<0.0001
Yes	1567 (80.6%)	1022 (89.2%)	536 (68.1%)	
No	378 (19.4%)	124 (10.8%)	251 (31.9%)	
Over the past six months, how often did you visit or consulted a general practitioner?^d^				<0.0001
Never	446 (22.9%)	108 (9.6%)	337 (46.0%)	
Once	405 (20.8%)	181 (16.1%)	221 (30.2%)	
2–4 times	545 (28.0%)	409 (36.4%)	129 (17.6%)	
≥ 5 times	475 (24.4%)	427 (38.0%)	45 (6.1%)	
Do not know	78 (4.0%)	27 (2.3%)	51 (6.5%)	
Reason of current visit^e^				
Sick/Unwell	729 (37.3%)	387 (33.6%)	338 (42.9%)	<0.0001
Medical check-up	422 (21.6%)	262 (22.7%)	258 (20.1%)	0.160
Prescription	877 (44.9%)	713 (61.8%)	157 (19.9%)	<0.0001
Referral	216 (11.0%)	140 (12.1%)	75 (9.5%)	0.070
Health certificate	106 (5.4%)	41 (3.6%)	64 (8.1%)	<0.0001
Second opinion	128 (6.5%)	77 (6.7%)	50 (6.3%)	0.771
Other	130 (6.6%)	43 (3.7%)	86 (10.9%)	<0.0001

^a^Based on 1950 cases for the variable presence of chronic disease

^b^Based on sample size of 1954; ^c^Based on a sample size of 1945; ^d^Based on a sample size 1949; ^e^Based on a sample size of 1955

**Table 3:** Participant assessment of GP accessibility according to presence of chronic disease

Variable	Overall^a^ n (%)	Patients with chronic disease^b^ n (%)	Patients without chronic disease^c^ n (%)	*p*-value
How long does it take to go from your home to the Primary care unit?				0.028
< 20 min	1335 (68.0%)	765 (66.2%)	559 (70.6%)	
20–40 min	433 (22.1%)	271 (23.4%)	160 (20.2%)	
40–60 min	127 (6.5%)	87 (7.5%)	40 (5.1%)	
> 1 h	56 (2.9%)	29 (2.5%)	27 (3.4%)	
Did you have to make an appointment for your visit to the GP?				0.007
Yes	523 (27.3%)	333 (29.4%)	183 (23.8%)	
No	1392 (72.7%)	800 (70.6%)	587 (76.2%)	
Was it easy to make an appointment?				0.117
Yes	474 (86.2%)	294 (84.2%)	172 (89.1%)	
No	76 (13.8%)	55 (15.8%)	21 (10.9%)	
How many days did you wait for today’s visit?				0.546
None	171 (32.4%)	107 (31.7%)	58 (32.2%)	
One day	143 (27.1%)	86 (25.4%)	56 (30.6%)	
2–7 days	125 (23.7%)	85 (25.1%)	40 (21.9%)	
> 7 days	89 (16.9%)	60 (17.8%)	28 (15.3%)	
Don’t know	25 (4.5%)	11 (3.2%)	13 (6.6%)	
How long did you have to wait to see your doctor when you arrived today?				<0.0001
< 15 min	553 (28.7%)	295 (25.7%)	258 (33.0%)	
15–30 min	598 (31.0%)	342 (29.8%)	256 (32.7%)	
30–45 min	378 (19.6%)	246 (21.5%)	132 (16.9%)	
45–60 min	184 (9.5%)	123 (10.7%)	61 (7.8%)	
> 1 h	203 (10.5%)	135 (11.8%)	68 (8.7%)	
Don’t know	12 (0.6%)	5 (0.4%)	7 (0.8%)	
It is difficult to see a GP in the evenings, at night and in the weekend?				0.509
Yes	421 (21.9%)	251 (22.0%)	170 (21.7%)	
No	1046 (54.4%)	642 (56.3%)	404 (51.6%)	
Don’t know	457 (23.8%)	248 (21.7%)	209 (26.7%)	

*p*-values report on significance between *t*-test comparison by presence of chronic disease

^a^Based on 1964 cases unless otherwise indicated; ^b^Based on 792 cases unless otherwise indicated; ^c^Based on 1158 cases unless otherwise indicated

### Patient experience questionnaire

Tables 3 and 4 present the results regarding participant’s experiences at the GP consultation at which they were surveyed for each of the 5 main dimensions assessed. We review main findings for each dimension below.

**Table 4:** Participant experience assessment by primary care domain overall and according to presence of chronic disease

Variable	% Overall sample *n* = 1964	% Patients with chronic disease *n* = 1158	% Patients w/out chronic disease *n* = 792	*p*-value*
Accessibility				
Opening hours are too restricted	28.8	30.8	25.7	0.025
If I need a home visit I can get one	71.2	73.6	67.4	0.009
The practice is close to where I live or work	81.5	80.1	83.7	0.048
I had to wait too long on the phone	11.4	10.7	12.6	0.248
The doctor did not take sufficient time	83.4	82.5	84.8	0.190
It was easy to make an appointment	86.2	84.2	89.1	0.117
Same or next day appointment	59.5	57.1	62.8	0.546
I am able to see the GP in the evening, at night/weekend	72.8	71.9	70.3	0.509
Continuity and Coordination				
The GP had my medical history available in front of him/her	51.2	59.0	39.5	<0.0001
The GP knows important information about my medical history	80.4	87.2	69.8	<0.0001
The GP knows my living conditions	65.4	71.7	55.6	<0.0001
During the past two years, the GP asked me about all the medications that I was taking	76.6	86.7	60.9	<0.0001
It is difficult to get a referral to a medical specialist from my GP	10.1	8.9	10.7	0.257
Communication and Patient-Centred Care				
The GP was polite	94.8	95.6	94.2	0.157
The GP listened carefully to me	92.5	93.1	91.6	0.233
The GP looked at me when I was talking	88.4	88.2	91.0	0.053
The GP asked me questions about my health problem	91.9	91.9	91.9	0.992
I could not really understand what the GP was trying to explain to me	88.4	86.1	91.9	<0.0001
Comprehensiveness				
The doctor asked me about other problems beyond that for which I came	74.3	76.5	71.4	0.011
The GP is not only addressing my medical problems, but is also able to help with my personal problems and concerns	80.7	85.1	73.8	<0.0001
During the past 12 months, a GP talked to me about how to stay healthy (diet, smoking, alcohol)	72.6	81.2	60.1	<0.0001
Patient Activation				
The GP included me in the decisions about my treatment	73.5	74.1	72.5	0.440
After today’s visit, I feel that I am able to deal better with my health problem than before	86.3	86.3	86.2	0.975

^a^Based on 1950 cases or the variable presence of chronic disease (yes/no)

#### Accessibility

Most participants live within 20 min of their GPs office. The majority of patients did not book an appointment for their clinic visit with more than 40% of respondents reported waiting more than 30 min to see the GP. Among the 27.3% of participants who reported that they had to schedule an appointment with their GP and most reported the appointment booking to be easy. Most patients felt the GP spent enough time with them. Twenty-two percent reported they were not able to access GPs office in the evening or weekend.

#### Continuity & coordination

Among patients who reporting having a personal GP, 9.2% were surveyed at an appointment with a different GP from the same practice/centre and 10.3% had a personal GP who was located in a practice other than that from which they were surveyed. Approximately half of patients sampled reported that the GP had their medical history available in front of them, although patients with chronic disease were significantly more likely to report the GP had their medical history available. Seventy-seven per cent of participants reported that within the last 2 years the GP conducted a medication reconciliation and participants with a chronic disease were significantly more likely to report receiving lifestyle advice and reported having their medication history documented compared to individuals without a chronic disease. Half of participants felt they were able to receive a referral to a medical specialist when appropriate via the GP.

#### Communication & patient-centred care

Almost 95% of participants felt the GP was polite and 93% reported that the GP listened carefully during the physical examination.

#### Comprehensiveness

Seventy-four percent of participants mentioned that the GP asked them about other problems, beyond that for which they came to clinic to address while 81% reported that the GP was very helpful also with personal problems and concerns. Eighty-one percent of patients with chronic disease reported receiving advice related to staying healthy (diet, smoking, alcohol), this fell to 60% among individuals without a chronic disease (*P* < 0.0001).

#### Patient involvement

Eighty-six percent of the participants identified that after the visit with the GP that they felt better able to deal with their health problem than before. However, only 74% of respondents reported that the GP included them in their decisions about treatment.

### Patient values questionnaire

Table 5 presents data regarding the proportion of patients who ranked each item as very important for each domain of the Patient Values Questionnaire. Patients identified knowing in advance which doctor they will see for their consultation, that the physician does not cancel their appointment, and that the doctor is prepared for the consultation was of significant importance. Participants reported aspects of the GP encounter that were very important to be that the GP listened to them attentively (68.8%), is calm (59.4%) and treats them as a person and not as medical problem (57.8%). During the consultation, the following factors were ranked as very important: the physician checks with patient to ensure they have understood everything communicated (67.1%) and the physician is respectful during the physical examination and does not interrupt them (46.8%). Patients identified the ability to be honest and to talk in detail with their physician about their health problem as very important. Most patients (64.7%) felt it was very important to be informed by their physician about what to do if something goes wrong with their treatment. There were no significant differences between respondents without a chronic disease and those with chronic disease in terms of values related to primary care. The only exception was that the doctor asking if they had any questions was identified as very important by a larger proportion of respondents without a chronic disease.

**Table 5:** Patient value survey responses by quality domain – Proportion of participants who ranked as very important

Variable	% Total *n* =219	% Without chronic disease *n* =102	% Patients with chronic disease *n* = 117	*p*-value
Accessibility				
The practice has extensive opening hours	43.6	41.9	45.5	0.071
I can get an appointment easily at this practice	47.7	45.3	50.5	0.666
I know how to get evening, night and weekend services	47.2	50.4	43.6	0.416
The practice is close to where I live or work	57.8	63.2	51.5	0.053
I have a short waiting time on the phone when I call this practice	38.1	38.5	37.6	0.322
The doctor does not give me the feeling to be under time pressure	59.4	58	61.2	0.632
The doctor offers me his/her telephone or email to contact for further questions	29.4	28.7	29.3	0.923
I will keep my appointment	34.7	30.9	38.3	0.268
Continuity and Coordination				
The doctor has my medical records at hand	42.1	41.1	46.9	0.144
The doctor knows important information about my medical background and health issues	57.9	59.0	62.4	0.714
The doctor knows about my living situation	28.4	27.7	29.2	0.811
The doctor has prepared for the consultation by reading my medical notes	29.4	29.7	29.3	0.950
The doctor gives me instructions on what to do when things go wrong	64.7	59.4	69.0	0.142
I don’t need to tell a receptionist or nurse details about my health problem	21.6	22.2	21.4	0.920
I inform the doctor how the treatment works out	43.0	40.0	45.1	0.450
I can see another doctor if I think it is necessary	29.8	29.3	30.5	0.854
Communication and Patient-Centered Care				
The doctor is polite	63.9	59.4	67.5	0.215
The doctor makes me feel welcome by making eye contact	49.8	47.5	52.1	0.497
The doctor listens attentively	68.8	69.3	68.1	0.849
The doctor is aware of my personal, social and cultural background	13.7	13.9	13.7	0.968
The doctor is not prejudiced because of my age, gender, religion or culture	35.2	34.7	35.9	0.848
The doctor treats me as a person and not as a medical problem	57.8	52.5	62.1	0.154
The doctor is respectful during physical examinations and does not interrupt me	46.8	41.6	50.9	0.172
The doctor takes me seriously	54.7	48.5	59.6	0.103
The doctor understand me	56.7	51.0	62.1	0.101
The doctor asks me if I have any questions about my health problems	46.1	38.6	52.2	0.046
The doctor asks how I prefer to be treated	22.5	20.7	24.5	0.544
The doctor avoids disturbances of the consultation by telephone calls etc.	22.5	26.7	19.0	0.172
The people at the reception desk are polite and helpful	36.1	35.7	35.8	0.992
I understand clearly what the doctor explains	67.1	66.0	67.8	0.776
Comprehensiveness				
The doctor asks about possible other problems besides the one I came in for	37.2	34.0	40.0	0.364
The doctor gives me additional information about my health problem	20.1	18.8	21.4	0.639
The doctor informs me about reliable sources of information e.g. websites	7.8	7.9	7.8	0.965
Psychosocial issues (for example personal worries) can be discussed if needed	32.3	29.3	29.9	0.921
The doctor gives all test results even if they show abnormalities	49.1	50.5	47.4	0.651
Patient Activation				
The doctor involves me in making decisions about treatment	37.7	34.0	41.2	0.277
I feel able to cope better with my health problem after the visit	57.1	50.0	62.9	0.056
I have prepared for the consultation by keeping a symptom diary or preparing questions	15.3	15.0	15.8	0.873
I tell the doctor what I want to discuss in this consultation	26.1	28.0	23.9	0.495
I am prepared to ask questions and take notes	15.3	19.0	11.4	0.120
I am honest and not feel embarrassed to talk about my health problem	48.6	49.0	48.7	0.967
I am open about my use of other treatments, such as self-medication or alternative	25.1	25.3	24.3	0.878
I adhere to the agreed treatment plan	37.2	38.0	36.8	0.850
I can bring a family member/friend to the consultation	18.5	17.8	19.3	0.781
I inform the doctor how the treatment works out	42.7	45.1	40.0	0.781

*p*-values report on significance comparison by presence of chronic disease

Table 6 summarizes the top 11 values of the primary care interactions that were identified by respondents as well as the corresponding assessment.

**Table 6:** Aspects rated very important by the majority of patients and the corresponding patient experience assessment scores

Top 11 values	% Who ranked as “very important”	% Positive patient experience rating
The doctor listens attentively	68.8	92.5
I understand clearly what the doctor explains	67.1	88.4
The doctor gives me instructions on what to do when things go wrong	64.7	-
The doctor is polite	63.9	94.8
The doctor does not give me the feeling to be under time pressure	59.4	83.4
The doctor knows important information about my medical history and health issues	57.9	80.4
The practice is close to where I live or work	57.8	81.5
The doctor treats me as a person and not as a medical problem	57.8	-
I feel able to cope better with my health problem after the visit	57.1	86.3
The doctor understand me	56.7	-
That the doctor takes me seriously	54.7	-

## Discussion

### Main findings of the study

Over the past several years, Greece has been attempting to reform national health care services with a focus on strengthening PHC delivery in an effort to meet the needs of the population and ensure the efficient use of public resources [4]. As part of these quality improvement efforts several system-level changes have been by the Greek government including the establishment of the National Organization for Healthcare Provision as a unified central healthcare insurer, the establishment of an electronic prescribing system, and the creation of a Primary Healthcare Network [4, 19]. Progress in terms of achieving this goal has however received significant criticism [4, 19, 20]. A 2013 report on the state of primary care in Greece reported that Greece lacks key characteristics of strong primary care and should be a priority of health care reform activities moving forward [4].

The QUALICOPC project provided a framework to study patient centeredness at the country level, by exploring to patient’s experiences and expectations as it relates to primary care health delivery [1, 17, 18] and by comparing responses among patient with and without chronic disease to determine if these groups of patients differ in terms of experience and values. Our study found, that overall most patients surveyed reported good rates of satisfaction with their experience in the health care system. This is a promising finding given that our study was conducted during the austerity period in Greece, which has had significant impact on funding for health care services and personnel. However, several areas for improvement were also identified, including issues of accessibility, patient-centeredness, patient involvement and continuity of care, which we discuss here.

Factors which were ranked as very important by the majority of study participants are summarized in Table 7. In terms of accessibility, patients have identified the distance of PHC offices from their home or work, and not feeling time pressure from the physician as being highly valued. Both of these items were ranked by more than 80% of respondents as being met. While not ranked as the most valued aspects of care by patients, long wait-times are being reported on the day of their visit to clinic, by most patients. In Greece most GPs do not typically book appointments, which may explain the long office wait times reported by patients in the present evaluation [4]. Wait times and proximity of primary care centres are quality indicators [16, 21]. The challenges documented in present study in terms of wait times and access to primary care in non-urban areas have been previously identified in formal assessment of the primary care system in Greece and are important areas for quality improvement [4]. Importantly, while not assessed directly in the present study, patient out of pocket payments have been shown to limit access to primary health care services, a pattern which would be further increased by the economic crisis in Greece [4, 15].

**Table 7:** Factors related to primary health care delivery that were ranked as most valued by patients surveyed

More than half of participants also indicated the following as very important:
• The doctor listens attentively
• I understand clearly what the doctor explains
• The doctor gives me instructions on what to do when things go wrong
• The doctor is polite
• The doctor does not give me the feeling to be under time pressure
• The doctor knows important information about my medical history and health issues
• The practice is close to where I live or work
• The doctor treats me as a person and not as a medical problem
• I feel able to cope better with my health problem after the visit
• The doctor understand me
• That the doctor takes me seriously
• That the doctor know when to refer me to a medical specialist
• That the doctor makes me feel welcome by making eye contact
• That the doctor is respectful during physical examinations and does not interrupt me
• That the doctor asks me if I have any questions
• That the doctor gives all test results even if they show abnormalities
• That I know which doctor will I see
• That I am honest and not feel embarrassed to talk about my health problem
• That I know which doctor will I see
• That the doctor asks me if I have any questions

Primary care providers scored well in many aspects related to the communication and patient-centered care domain. In terms of areas for improvement, providers had moderate scores in the assessment of the patient’s living conditions, and discussing problems beyond the reason for the current visit. These two factors were scored significantly lower by patients who did not have a chronic disease as compared to individuals with chronic conditions. Additionally significantly fewer respondents with a chronic disease indicated that the GP assisted in addition to addressing the patients’ health problems with personal problems and concerns. The focus on urgent care issues and lack of comprehensive approach to family or community-based health care delivery is highlighted by this data - an issue that has been identified by other groups [4, 16, 21].

Patient-involvement in decision making about their treatment plan was scored moderately. Interestingly this variable did not differ between patients with and without a chronic disease. Approximately 15% of patients left the GPs office not feeling better prepared to deal with their medical condition, the reasons for this should be examined as an area for future research.

Several areas of improvement were identified in terms of the continuity of care received by patients. Importantly, only half of patient’s surveyed reported the GP had their medical history available in front of them. Availability of medical history records was 60% among patients with chronic disease and 40% among individuals who did not have a chronic disease. It is possible that this finding is related to the lack of an effective appointment booking system as was the lack of a comprehensive electronic medical records system [4]. Although certain efforts have been made in the recent years, primary care physicians currently only have electronic access to a record of patient’s prescriptions. Respondents with a chronic disease were significantly more likely to report receiving advice related to staying healthy (diet, smoking, alcohol), compared to individuals without a chronic disease (81% vs. 60%; *p* < 0.0001). All variables related to continuity and coordination were scored significantly lower among patients without a chronic condition, when compared to individuals with chronic disease.

Data from the present study is consistent with previous reports regarding the weaknesses of PHC system in Greece including the lack of integration across the health care system, limited use of multidisciplinary teams, poor coordination and continuity of care, as well as, a lack of focus on disease prevention and health promotion [4, 16, 22–25]. Using the quality standards set out by the QUALICOPC study group, Greece has scored low across all domains assessed with the exception of the continuity domain where a moderate ranking was received [17].

### Implications to practice and policy

Data gathered will assist with documenting the patient perspective and may be used to improve the quality of PHC services in Greece through an increased focus on patient-centered approaches especially in a period where a primary care reform is presently being examined by the current government and Greece struggles to develop a high quality system to respond to the population health needs. Study findings may also have application to other national health care systems and policy in countries with similar context and infrastructure.

Countries that have focused on strengthening PHC have emphasized the importance of patient-centered practices [17]. Internationally, quality improvement efforts in the primary care setting have emphasized issues such as patients with multiple co-morbidities, integrated primary care delivery, and workforce and technology (e.g. e-Health) [16, 26–28]. Studies have shown positive correlations between the patient-centered approach and patient experiences such as patient satisfaction [12, 29], patient recall of the content of the health care services [30], patient adherence [30], patient health outcomes [8, 12, 31, 32], health care utilization [8, 30] and provider satisfaction [30]. The financial crisis in Greece necessitates a closer look at proven models, such as the patient-centered model of care, for improving population health, reducing inequalities, and spending. This is a fertile area for GPs and the primary care health care system to invest energy and international experience and collaborations can serve to guide these efforts [33].

Training programs, which engage GP’s in more patient-centered communication have been shown by to lead to an increased likelihood that the interaction would be more closely aligned with the patient’s psychosocial needs and improve patient’s compliance and outcomes [30]. Given this evidence, we would recommend government as well as medical schools within Greece look at strengthening the emphasis on patient-centered care within medical school curricula and continuing medical education programs. A recommendation that was brought forward in a recent report examining primary health care in Europe [16, 21, 34].

Our study identified a significant gap in primary care provider’s access to patient medical history, a factor, which was highly valued by patients surveyed. These results suggest that policy-makers should continue efforts to improve PHC services by adopting the Electronic health Record (EHR), which is expected to improve all six dimensions of quality care identified by the Institute of Medicine (safety, timeliness, effectiveness, efficiency, equity, and patient-centeredness) [35]. Unfortunately, at present, only the prescription part of the EHR is operational and there is feedback from providers that this tool is time consuming. International experience based on episode of care and ICPC-2 classification could be used to inform quality improvement activities related to the introduction of EHRs in Greece [36, 37].

Our study also documented several gaps in care for individuals who did not have a diagnosed chronic condition, in particular in terms of comprehensiveness of care. There were several important differences documented between respondents without a chronic disease and those with a chronic condition in terms of their experience with the primary care system. This data may highlight a lack of attention to preventative care within the national health care system in Greece and is consistent with previous reports [4, 16, 38]. The financial constraints has meant several areas of international importance in the field of primary care are not on the agendas of GPs in Greece including frailty, chronic disease prevention, self-management, and multi-morbidity [33]. Given the high rates of chronic disease documented in Greece, the delay in diagnosis of chronic diseases such as COPD and lung cancer, and increased focus on preventative medicine and chronic disease-management within the primary care system is recommended [39]. Highly cost-effective interventions are available and have been tested in other countries, as well as in Greece, which should be implemented as part of the national primary care system [40]. Likewise, the role of primary care and public health nurses must be urgently discussed and there is evidence that the capacity of nurses serving in the Greek primary care is underutilized [41–43]. Finally, the development of quality standards in PHC services which are informed by patient-centered models of care are particularly important during the present austerity period in Greece.

### Limitations

The present study has some methodological limitations that should be understood when interpreting findings. First the recruitment of GP’s was not via random selection but rather used a local champion who assisted with GP recruitment in each of the seven health regions in Greece and as such may not necessarily be representative of all GPs in Greece. Our sample primarily represented the views of GPs who serve in the public sector. The number of private practice GPs included in the study was small (*n* = 17), and as such was not sufficiently representative of all private practices in Greece, thus we are not certain to what extent the views of GPs serving in the private sector differ. Given the study required patients to read and/or write in Greek our findings do not represent this patient population. The study results may be subject to observer bias, which may have resulted in providers increasing their performance during the period in which survey data was collected. Additionally, the nature of the present survey required that patients be able to read in Greek and as such is not representative of patient’s who are unable to read and may in particular not be representative of lower income individuals, individuals with disabilities, and those whose mother tongue is something other than Greek. Furthermore, although the sample size of the study is relatively large, there is no data available regarding the non-responding patients thus inducing a potential source of selection bias. The present study used a previously validated tool to assess patient-valued and experiences but may not assess all aspects of care that are valued by patients. Finally, as has been reported by the QUALICOPC authors previously patient values may be a reflection of their experience to date in the primary health care system. For example in low performing health care system patients may have lower expectations about health care delivery and rank importance differently. As such information from patient value assessments should be considered one but not the only source of information for informing quality improvement activities [17].

## Conclusions

In summary, our study presents data about patient’s experiences and values across the 5 domains of primary health care delivery: Accessibility, Continuity & Coordination, Communication & Patient-Centred Care, Patient Activation, Comprehensiveness. A positive finding of the survey is that most of the patients (especially patients with chronic disease, who visit primary care unit more regularly) surveyed report that their health needs are being met. Several areas for quality improvement related in particular to access, comprehensiveness, and prevention have been identified which can be used to inform quality improvement activities to strengthen primary care delivery in Greece during a period where national health care reform is under discussion.

## Abbreviations

GP: General practitioner
ELEGEIA: Greek Association of General Practitioners
PHC: Primary Health Care
NIVEL: Netherlands Institute for Health Services Research
